# The synthesis and crystal structure of 2-(chloro­selan­yl)pyridine 1-oxide: the first monomeric organoselenenyl chloride stabilized by an intra­molecular secondary Se⋯O inter­action

**DOI:** 10.1107/S2056989016018946

**Published:** 2016-11-30

**Authors:** Rizvan K. Askerov, Zhanna V. Matsulevich, Galina N. Borisova, Svetlana A. Zalepkina, Vasiliy F. Smirnov, Maria M. Grishina, Pavel V. Dorovatovskii, Alexander V. Borisov, Victor N. Khrustalev

**Affiliations:** a Department of Chemistry, Baku State University, 23 Z. Khalilov St., Baku, AZ-1148, Azerbaijan; b R.E. Alekseev Nizhny Novgorod State Technical University, 24 Minin St., Nizhny Novgorod, 603950, Russian Federation; c N.I. Lobachevsky State University of Nizhny Novgorod, 23 Gagarin Prosp., Nizhny Novgorod, 603950, Russian Federation; d Inorganic Chemistry Department, Peoples’ Friendship University of Russia, 6 Miklukho-Maklay St., Moscow, 117198, Russian Federation; e National Research Center "Kurchatov Institute", 1 Acad. Kurchatov Sq., Moscow, 123182, Russian Federation; f X-Ray Structural Centre, A.N. Nesmeyanov Institute of Organoelement Compounds, Russian Academy of Sciences, 28 Vavilov St., B–334, Moscow 119991, Russian Federation

**Keywords:** crystal structure, synchrotron radiation, organoselenenyl chloride, intra­molecular stabilization, secondary inter­actions

## Abstract

The title compound, C_5_H_4_ClNOSe, is the product of the reaction of sulfuryl chloride and 2-selanyl-1-pyridine 1-oxide in di­chloro­methane. The mol­ecule has an almost planar geometry (r.m.s. deviation = 0.012 Å), and its mol­ecular structure is stabilized by an intra­molecular secondary Se⋯O inter­action of 2.353 (3) Å, closing a four-membered N—C—Se⋯O ring. The title compound represents the first monomeric organoselenenyl chloride stabilized intra­molecularly by an inter­action of this type. The non-valent attractive Se⋯O inter­action results in a substantial distortion of the geometry of the *ipso*-carbon atom. The *endo*-cyclic N—C—Se [102.1 (3)°] and *exo*-cyclic C—C—Se [136.9 (3)°] bond angles deviate significantly from the ideal value of 120° for an *sp* ^2^-hybridized carbon atom, the former bond angle being much smaller than the latter. In the crystal, mol­ecules are linked by C—H⋯O hydrogen bonds, forming zigzag chains propagating along [010]. The chains, which stack along the *a-*axis direction, are linked by offset π–π inter­actions [inter­centroid distance = 3.960 (3) Å], forming corrugated sheets parallel to the *ab* plane.

## Abstract 2

2-(Chloro­selan­yl)pyridine 1-oxide represents the first monomeric organoselenenyl chloride stabilized by an intra­molecular secondary Se⋯O inter­action.

## Chemical context   

Organoselenenyl halides *R*Se*X* (*X* = Cl, Br) play an important role in modern organic synthesis and are used as reagents for the functionalization of many classes of compounds, including organoselenium compounds with a broad spectrum of biologi­cal activities (Ranganathan *et al.*, 2004 ▸; Selvakumar *et al.*, 2010 ▸, 2011 ▸; Ninomiya *et al.*, 2011 ▸; Singh & Wirth, 2011 ▸; Zade & Singh, 2014 ▸; Elsherbini *et al.*, 2016 ▸). An essential aspect of the chemistry of selenenyl halides is the factors responsible for the stability of these reagents (Coles, 2006 ▸; Mukherjee *et al.*, 2010 ▸; Nakanishi *et al.*, 2013 ▸; Takaluoma *et al.*, 2015 ▸). Recently, we have developed a new effective method for the stabilization of heteroarenselenenyl and -tellurenyl chlorides by the transformation of them to T-shaped zwitterionic adducts with hydro­chloric acid (Khrustalev *et al.*, 2012 ▸, 2014 ▸, 2016 ▸). Moreover, we have established another stabilization method of heteroarenselenenyl and -tellurenyl chlorides by inter­molecular secondary Ch⋯N (Ch = Se, Te) inter­actions with the formation of dimers (Borisov *et al.*, 2010*a* ▸,*b* ▸,*c* ▸; Khrustalev *et al.*, 2016 ▸). Herein, we report on the synthesis and structural characterization of the first monomeric 2-(chloro­selan­yl)pyridine 1-oxide stabilized by an intra­molecular secondary Se⋯O inter­action.
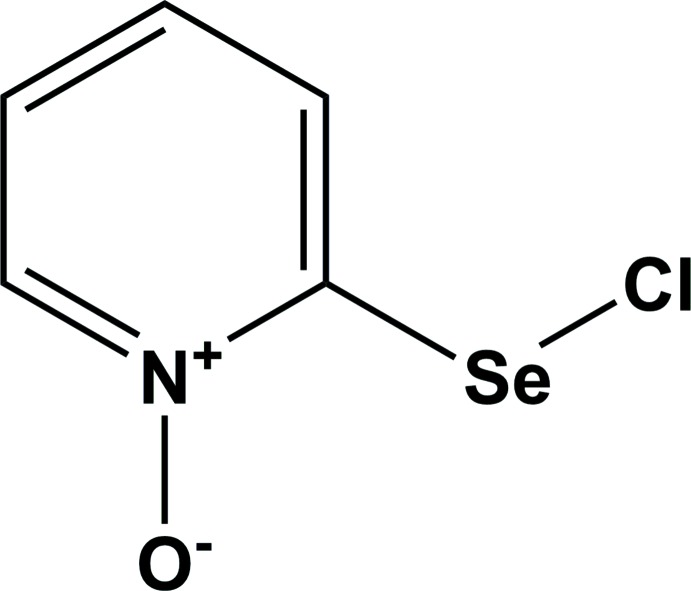


## Structural commentary   

**Figure 1:**
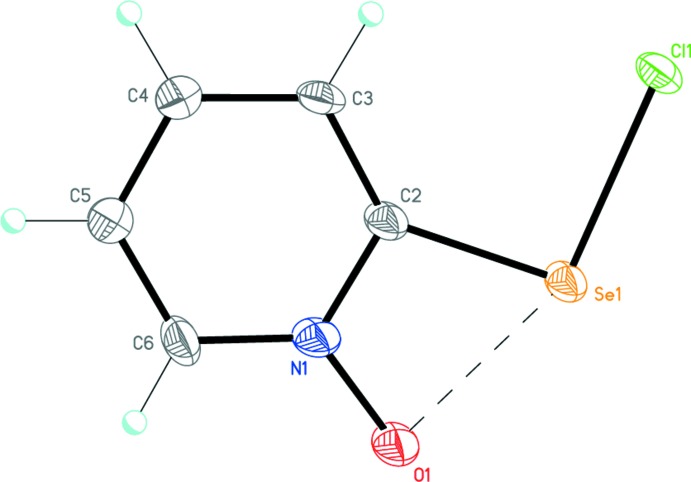
The mol­ecular structure of the title compound, with atom labelling and displacement ellipsoids drawn at the 50% probability level. The dashed line indicates the intra­molecular secondary attractive Se1⋯O1 inter­action.

The title compound, Fig. 1 ▸, is the product of the reaction of sulfuryl chloride and 2-selanyl-1-pyridine 1-oxide in di­chloro­methane. It has an almost planar geometry (r.m.s. deviation = 0.012 Å), and its mol­ecular structure is stabilized by an intra­molecular secondary Se1⋯O1 inter­action of 2.353 (3) Å, closing the four-membered N1—C2—Se1⋯O1 ring (Fig. 1 ▸). The non-valent attractive Se1⋯O1 inter­action results in the substantial distortion of the geometry of the *ipso*-C2 carbon atom. The *endo*-cyclic N1—C2—Se1 [102.1 (3)°] and *exo*-cyclic C3—C2—Se1 [136.9 (3)°] bond angles deviate significantly from the ideal value of 120° for an *sp* ^2^-hybridized carbon atom, the former angle being much smaller than the latter. The title compound represents the first monomeric organoselenenyl chloride stabilized intra­molecularly by an inter­action of this type. Previously, the analogous stabilization of monomeric organoselenenyl chlorides by intra­molecular secondary Se⋯S (Tiecco *et al.*, 2006 ▸) and Se⋯N (Panda *et al.*, 1999 ▸; Klapötke *et al.*, 2004 ▸; Kulcsar *et al.*, 2007 ▸; Pöllnitz *et al.*, 2011 ▸) inter­actions have been reported.

## Supra­molecular features   

**Figure 2:**
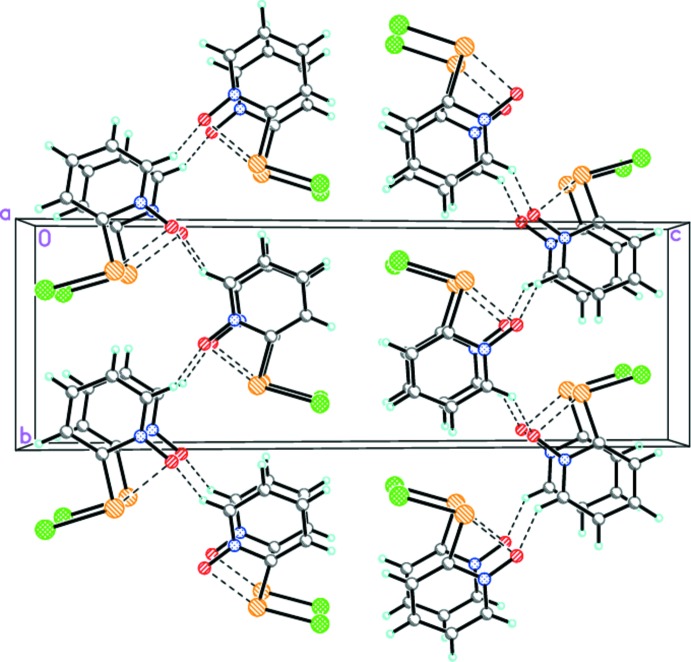
The crystal packing of the title compound viewed along the *a* axis. The intra­molecular secondary Se⋯O inter­actions and the inter­molecular C—H⋯O hydrogen bonds are shown as dashed lines (see Table 1 ▸).

In the crystal, mol­ecules are linked by C—H⋯O hydrogen bonds (Table 1 ▸ and Fig. 2 ▸), forming zigzag chains propagating along the *b-*axis direction. The chains stack along the *a-*axis direction and are linked by offset π–π inter­actions, forming corrugated sheets parallel to the *ab* plane [*Cg*⋯*Cg* ^i,ii^ = 3.960 (3) Å, *Cg* is the centroid of the N1/C2–C6 ring, inter­planar distances = 3.590 (2) Å, slippages = 1.671 Å, symmetry codes: (i) *x* − 1, *y*, *z*; (ii) *x* + 1, *y*, *z*].

## Synthesis and crystallization   

**Figure 3:**
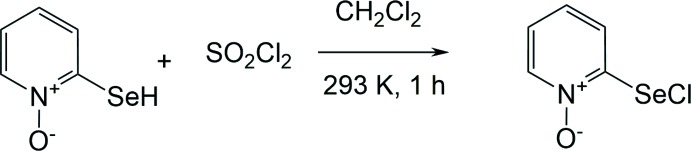
The synthesis of the title compound; the reaction of 2-selanyl-1-pyridine 1-oxide with sulfuryl chloride in di­chloro­methane.

The synthesis of the title compound is illustrated in Fig. 3 ▸. It was synthesized according to the procedure described previously by Borisov *et al.* (2010*a* ▸,*b* ▸,*c* ▸). A solution of sulfuryl chloride (0.27 g, 2 mmol) in di­chloro­methane (15 ml) was added to a solution of 2-selanyl-1-pyridine 1-oxide (0.35 g, 2 mmol) in di­chloro­methane (20 ml) at 293 K. After one h it was filtered to give the title compound (yield 0.33 g, 80%). The filtrate was evaporated *in vacuo* and recrystallization of the residue from di­chloro­methane solution gave an additional 0.06 g (15%) of the title compound. Colourless prismatic crystals of the title compound were obtained after recrystallization of the crude product from di­chloro­methane (m.p. 433–435 K). IR (KBr, cm^−1^), ν 1617, 1462, 1423, 1254, 1151, 836, 748, 621. ^1^H NMR (DMSO-*d*_6_, 300 MHz, 300 K): δ = 8.28 (*d*, 1H, ^3^ *J* = 5.9, H6); 7.52 (*d*, 1H, ^3^ *J* = 7.3, H3); 7.43 (*dd*, 1H, ^3^ *J* = 7.8, ^3^ *J* = 7.3, H4); 7.30 (*dd*, 1H, ^3^ *J* = 7.8, ^3^ *J* = 5.9, H5). Analysis calculated for C_5_H_4_ClNOSe: C 24.81; H 1.93; N 6.72. Found: 24.43; H 1.83; N 6.65.

## Refinement   

Crystal data, data collection and structure refinement details are summarized in Table 2 ▸. The C-bound H atoms were placed in calculated positions and refined as riding: C—H = 0.95 Å with *U*_iso_(H) = 1.2*U*_eq_(C).

## Supplementary Material

Crystal structure: contains datablock(s) global, I. DOI: 10.1107/S2056989016018946/su5337sup1.cif

Structure factors: contains datablock(s) I. DOI: 10.1107/S2056989016018946/su5337Isup2.hkl

CCDC reference: 1519449

Additional supporting information: crystallographic information; 3D view; checkCIF report

## Figures and Tables

**Table 1:** Hydrogen-bond geometry (Å, °)

*D*—H⋯*A*	*D*—H	H⋯*A*	*D*⋯*A*	*D*—H⋯*A*
C6—H6⋯O1^i^	0.95	2.34	3.101 (6)	137

Symmetry code: (i) 
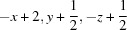
.

**Table 2:** Experimental details

Crystal data	
Chemical formula	C_5_H_4_ClNOSe
*M*_r_	208.50
Crystal system, space group	Monoclinic, *P*2_1_/*c*
Temperature (K)	100
*a*, *b*, *c* (Å)	3.9601 (8), 7.5102 (15), 22.350 (5)
β (°)	94.32 (3)
*V* (Å^3^)	662.8 (2)
*Z*	4
Radiation type	Synchrotron, λ = 0.96990 Å
μ (mm^−1^)	13.68
Crystal size (mm)	0.05 × 0.03 × 0.03

Data collection	
Diffractometer	Rayonix SX-165 CCD
Absorption correction	Multi-scan (*SCALA*; Evans, 2006 ▸)
*T*_min_, *T*_max_	0.550, 0.660
No. of measured, independent and observed [*I* > 2σ(*I*)] reflections	5526, 1310, 1121
*R*_int_	0.083
(sin θ/λ)_max_ (Å^−1^)	0.636

Refinement	
*R*[*F* ^2^ > 2σ(*F* ^2^)], *wR*(*F* ^2^), *S*	0.074, 0.175, 1.01
No. of reflections	1310
No. of parameters	83
H-atom treatment	H-atom parameters constrained
Δρ_max_, Δρ_min_ (e Å^−3^)	1.26, −1.58

Computer programs: *Automar* (MarXperts, 2015 ▸), *iMosflm* (Battye *et al.*, 2011 ▸), *SHELXS97* and *SHELXTL* (Sheldrick, 2008 ▸) and *SHELXL2014/6* (Sheldrick, 2015 ▸).

## supplementary crystallographic information

### Crystal data

**Table:**

C_5_H_4_ClNOSe	*F*(000) = 400
*M**_r_* = 208.50	*D*_x_ = 2.089 Mg m^−^^3^
Monoclinic, *P*2_1_/*c*	Synchrotron radiation, λ = 0.96990 Å
*a* = 3.9601 (8) Å	Cell parameters from 600 reflections
*b* = 7.5102 (15) Å	θ = 5.0–35.0°
*c* = 22.350 (5) Å	µ = 13.68 mm^−^^1^
β = 94.32 (3)°	*T* = 100 K
*V* = 662.8 (2) Å^3^	Prism, colourless
*Z* = 4	0.05 × 0.03 × 0.03 mm

### Data collection

**Table:**

Rayonix SX-165 CCD diffractometer	1121 reflections with *I* > 2σ(*I*)
/f scan	*R*_int_ = 0.083
Absorption correction: multi-scan (*SCALA*; Evans, 2006)	θ_max_ = 38.1°, θ_min_ = 5.0°
*T*_min_ = 0.550, *T*_max_ = 0.660	*h* = −4→4
5526 measured reflections	*k* = −9→9
1310 independent reflections	*l* = −28→28

### Refinement

**Table:**

Refinement on *F*^2^	Secondary atom site location: difference Fourier map
Least-squares matrix: full	Hydrogen site location: inferred from neighbouring sites
*R*[*F*^2^ > 2σ(*F*^2^)] = 0.074	H-atom parameters constrained
*wR*(*F*^2^) = 0.175	*w* = 1/[σ^2^(*F*_o_^2^) + (0.06*P*)^2^ + 1.6*P*] where *P* = (*F*_o_^2^ + 2*F*_c_^2^)/3
*S* = 1.01	(Δ/σ)_max_ = 0.001
1310 reflections	Δρ_max_ = 1.26 e Å^−^^3^
83 parameters	Δρ_min_ = −1.58 e Å^−^^3^
0 restraints	Extinction correction: SHELXL, Fc^*^=kFc[1+0.001xFc^2^λ^3^/sin(2θ)]^-1/4^
Primary atom site location: difference Fourier map	Extinction coefficient: 0.054 (3)

### Special details

**Table:**

Geometry. All esds (except the esd in the dihedral angle between two l.s. planes) are estimated using the full covariance matrix. The cell esds are taken into account individually in the estimation of esds in distances, angles and torsion angles; correlations between esds in cell parameters are only used when they are defined by crystal symmetry. An approximate (isotropic) treatment of cell esds is used for estimating esds involving l.s. planes.

### Fractional atomic coordinates and isotropic or equivalent isotropic displacement parameters (Å^2^)

**Table:**

	*x*	*y*	*z*	*U*_iso_*/*U*_eq_	
Se1	0.51523 (13)	0.26936 (7)	0.34782 (2)	0.02716 (17)	
Cl1	0.4514 (3)	0.18592 (14)	0.44303 (4)	0.0331 (3)	
O1	0.6571 (9)	0.4577 (4)	0.26942 (12)	0.0347 (8)	
N1	0.7603 (10)	0.5643 (5)	0.31523 (14)	0.0290 (8)	
C2	0.7093 (11)	0.4927 (5)	0.36941 (16)	0.0266 (9)	
C3	0.7969 (12)	0.5838 (6)	0.42160 (17)	0.0301 (10)	
H3	0.7578	0.5342	0.4596	0.036*	
C4	0.9449 (14)	0.7515 (6)	0.4172 (2)	0.0334 (13)	
H4	1.0115	0.8173	0.4524	0.040*	
C5	0.9941 (12)	0.8213 (7)	0.36099 (19)	0.0343 (12)	
H5	1.0906	0.9365	0.3579	0.041*	
C6	0.9047 (14)	0.7257 (5)	0.3095 (2)	0.0317 (12)	
H6	0.9436	0.7720	0.2710	0.038*	

### Atomic displacement parameters (Å^2^)

**Table:**

	*U*^11^	*U*^22^	*U*^33^	*U*^12^	*U*^13^	*U*^23^
Se1	0.0427 (4)	0.0202 (3)	0.0200 (3)	−0.00320 (19)	0.0117 (3)	−0.00209 (16)
Cl1	0.0522 (7)	0.0277 (5)	0.0208 (4)	−0.0077 (5)	0.0122 (4)	0.0037 (4)
O1	0.058 (2)	0.0284 (15)	0.0190 (12)	−0.0061 (14)	0.0139 (13)	−0.0045 (12)
N1	0.044 (2)	0.0265 (17)	0.0177 (14)	0.0022 (16)	0.0106 (13)	−0.0035 (13)
C2	0.041 (2)	0.0217 (19)	0.0183 (16)	0.0003 (18)	0.0111 (15)	−0.0008 (14)
C3	0.049 (3)	0.028 (2)	0.0144 (16)	−0.0006 (19)	0.0089 (16)	0.0008 (15)
C4	0.049 (3)	0.027 (2)	0.024 (2)	−0.0035 (19)	0.004 (2)	−0.0028 (15)
C5	0.051 (3)	0.026 (2)	0.0267 (19)	−0.002 (2)	0.0033 (19)	−0.0008 (19)
C6	0.048 (3)	0.0180 (18)	0.030 (2)	0.0009 (18)	0.009 (2)	0.0062 (15)

### Geometric parameters (Å, º)

**Table:**

Se1—C2	1.892 (4)	C3—H3	0.9500
Se1—Cl1	2.2506 (11)	C4—C5	1.389 (7)
O1—N1	1.339 (4)	C4—H4	0.9500
N1—C6	1.350 (6)	C5—C6	1.381 (6)
N1—C2	1.354 (5)	C5—H5	0.9500
C2—C3	1.374 (6)	C6—H6	0.9500
C3—C4	1.395 (6)		
			
C2—Se1—Cl1	94.48 (11)	C5—C4—C3	119.6 (4)
O1—N1—C6	124.8 (3)	C5—C4—H4	120.2
O1—N1—C2	112.9 (3)	C3—C4—H4	120.2
C6—N1—C2	122.3 (4)	C6—C5—C4	120.9 (4)
N1—C2—C3	121.0 (4)	C6—C5—H5	119.6
N1—C2—Se1	102.1 (3)	C4—C5—H5	119.6
C3—C2—Se1	136.9 (3)	N1—C6—C5	118.1 (4)
C2—C3—C4	118.0 (4)	N1—C6—H6	120.9
C2—C3—H3	121.0	C5—C6—H6	120.9
C4—C3—H3	121.0		
			
O1—N1—C2—C3	−179.4 (4)	Se1—C2—C3—C4	178.9 (4)
C6—N1—C2—C3	1.4 (7)	C2—C3—C4—C5	0.9 (7)
O1—N1—C2—Se1	0.7 (4)	C3—C4—C5—C6	−1.3 (8)
C6—N1—C2—Se1	−178.5 (4)	O1—N1—C6—C5	179.2 (4)
Cl1—Se1—C2—N1	−179.0 (3)	C2—N1—C6—C5	−1.7 (7)
Cl1—Se1—C2—C3	1.0 (5)	C4—C5—C6—N1	1.6 (8)
N1—C2—C3—C4	−1.0 (7)		

### Hydrogen-bond geometry (Å, º)

**Table:**

*D*—H···*A*	*D*—H	H···*A*	*D*···*A*	*D*—H···*A*
C6—H6···O1^i^	0.95	2.34	3.101 (6)	137

Symmetry code: (i) −*x*+2, *y*+1/2, −*z*+1/2.
